# Cephalometric assessment of craniofacial morphology in Japanese male patients with obstructive sleep apnea–hypopnea syndrome

**DOI:** 10.1111/j.1479-8425.2012.00539.x

**Published:** 2012-07

**Authors:** Yujiro Takai, Yoshihiro Yamashiro, Daisuke Satoh, Kazutoshi Isobe, Susumu Sakamoto, Sakae Homma

**Affiliations:** 1 Department of Respiratory Medicine, Toho University School of MedicineTokyo; 2 Ota General Hospital Sleep Disorder CenterKawasaki City, Kanagawa, Japan

**Keywords:** apnea–hypopnea index, craniofacial morphology, jaw soft tissue ratio, mandibular plane – hyoid bone, obstructive sleep apnea–hypopnea

## Abstract

Craniofacial morphological anomalies can be divided into two principal categories: skeletal anomalies and soft tissue anomalies. This study examined the hypothesis that the assessment of indices representing both skeletal and soft tissue can be used to appropriately identify the risk factor of obstructive sleep apnea–hypopnea syndrome (OSAHS). 232 suspected OSAHS male patients were examined with polysomnography and divided into two groups (202 males with OSAHS and 30 male controls without OSAHS). Cephalometric analysis was performed on all patients to evaluate craniofacial morphological anomalies. The measurement sites were as follows: skeletal morphology; soft tissue morphology; mixed morphology including mandibular plane to hyoid bone (MP-H); and jaw soft tissue (JS) ratio; a novel ratio we defined, between the area of jaw and area of tongue with soft palate. JS ratio increased with AHI as well as MP-H. MP-H and JS ratio showed significant but weak correlation with apnea–hypopnea index. JS ratio was significantly associated with an increased risk for severe OSAHS, even after adjusting age and BMI, its odds ratio was the greatest among these variables. These results showed that mixed craniofacial, skeletal and soft tissue morphology are correlated with AHI, and JS ratio may be a useful parameters to explain the characteristics of OSAHS in male patients.

## INTRODUCTION

Obstructive sleep apnea–hypopnea syndrome (OSAHS) is a multifactorial disorder. Previous studies suggest obesity,^1^–^3^ age,^4^ and gender^5^,^6^ are important factors of OSAHS. Craniofacial morphology is another important factor that could contribute to collapsing upper airways during sleep.^7^ Craniofacial morphologic abnormalities fall into two principal categories: skeletal anomalies, such as a small jaw; and soft tissue anomalies, such as enlargement of the soft palate or tongue area. Skeletal morphology is determined by genetic and developmental factors, whereas the morphology of soft tissue such as the tongue is related to body mass index (BMI).^8^ Racial differences in craniofacial morphology and associated abnormalities are known, and studies suggest that the consequences of craniofacial morphologic abnormalities are more severe in Asian populations than that of Caucasians with the same range of BMI or the degree of obesity.^9^,^10^ Thus craniofacial morphologic abnormalities as a cause of OSAHS may be greater in Asian populations. Craniofacial morphology as a risk assessment of OSAHS should be determined based on a balance between the jaw as a container and soft tissue as its content.^11^

Most studies^1^,^8^,^12^–^16^ relating skeletal and soft tissue morphology by cephalometry or magnetic resonance imaging (MRI) use the measurements of lengths and angles of each variable specific to the method at the nasopharynx area, whereas few studies examine both hard and soft tissue.^17^–^19^ Watanabe *et al*. reported by using cephalometry that mandibular and maxillary length (jaw size) between the length from mandible plane to hyoid bone (MP-H; represents soft tissue) were inversely correlated, and the association between the oxygen desaturation index (ODI) and MP-H.^11^ Tuiki *et al*. report, by measuring the size of jaw as a rectangule using cephalometry, that the tongue is significantly larger in subjects with larger maxillomandible dimensions; OSAHS patients have a significantly larger tongue for a given maxillomandible size than non-OSA subjects.^20^ Both studies suggest that the balance between the size of the jaws and the amount of soft tissue is related to the severity of OSAHS.

We hypothesize that a ratio of maxillomandible area (skeletal morphology), and tongue and soft palate area (soft tissue morphology) may identify risk factors of OSAHS effectively. Thus we evaluated validity of cephalometric variables including jaw soft tissue (JS) ratio, a novel index we defined, in order to explain the characteristics of OSAHS among Japanese male patients.

## METHODS

### Study subjects

Two hundred and seventy-two male patients visited to the hospital who were suspected OSAHS, and underwent polysomnography between February 2003 and December 2007. In these patients, twenty patients with tonsillar hypertrophy that was greater than II degrees according to the classification of Friedman *et al*. were excluded from this study.^21^ Two hundred and two patients (the mean values ± SD were: 48.8 ± 13.7 years in age (range, 19–86 years); 26.1 ± 4.4 kg/m^2^ in BMI; and 38.3 ± 27.6 events/hr in apnea–hypopnea index (AHI)) were diagnosed as OSAHS, and 30 patients (Age 38.3 ± 14.1 years, BMI 23.3 ± 3.0 kg/m^2^ AHI 2.6 ± 1.7 events/h) whose AHI were less than 5, were selected as control subjects. All subjects were Japanese (Asian) and all eligible patients were enrolled consecutively. Presenting symptoms of all subjects were either or both snoring and daytime sleepiness. OSAHS was defined as an AHI of 5 events per hour or more under polysomnography. All subjects were Japanese (Asian) and all eligible patients were enrolled consecutively. The study was approved by the Toho University School of Medicine Ethical Committee, and informed consent was obtained from each patient.

### Demographic characteristics

We determined the subject ethnicity by self-identification. BMI was calculated from the height and the weight of the subject according to the formula: weight in kilograms/(height in meters)^2^. We defined BMI < 25 as normal, and BMI ≥ 25 as obesity.^22^

### Polysomnography

Standard overnight polysomnography was performed with a continuous polygraphic form recording the following parameters: electroencephalography, electro-oculography, submental electromyography, and electrocardiography from surface leads; oronasal airflow signal from nasal pressure sensor; respiratory effort signals from thoracic and abdominal impedance belts; oxyhemoglobin level from pulse oximetry; snoring using a tracheal microphone, and body position changes during sleep. Polysomnography records were scored manually according to standard criteria.^23^,^24^

### Cephalometry

**Figure 1:**
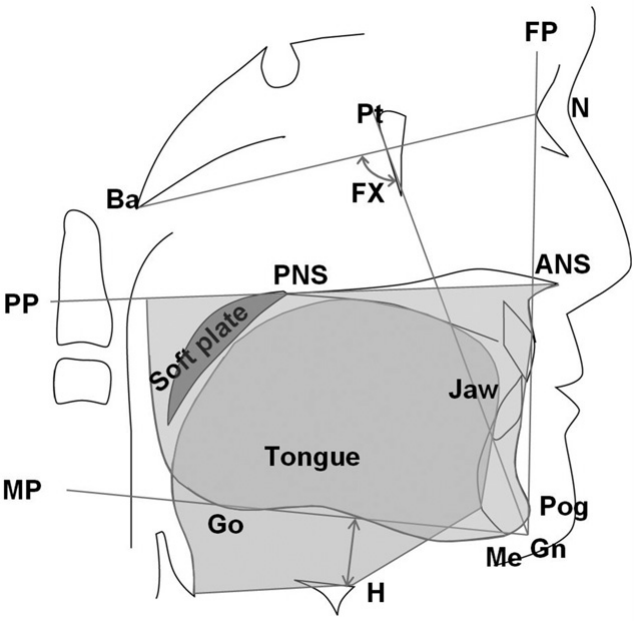
Cephalometric point of skeletal, soft tissue and mixed morphology. ANS, anterior nasal spine; Ba, basion; FP, facial plane; Gn, gnathion; Go, gonion; H, hyoid; Me, menton; MP, mandibular plane; MP-H, mandibular plane – hyoid; N, nasion; PNS, posterior nasal spine; Pog, pogonion; PP, palatal plane; Pt, pterygoid point. The following angles in degrees and dimensions in millimeters were measured: FX: facial axis. The following cross-sectional areas are in cm^2^: soft palate area: outlined by the soft palate surface; tongue area: outlined by the dorsum of the tongue surface and lines that connect tongue tip, spina mentalis, hyoid, and base of epiglottis; jaw area: under the PP and outlined lines that connect ANS, subspinale, prosthion, infradentale, supramentale, Pog, Me and Go.

Cephalometric analysis (DHF-158H II, HITACHI, Tokyo, Japan) was performed for each patient during their first visits. We used sitting position as it provides a natural head position. Standardized lateral cephalograms were taken as a part of the normal protocol for evaluation of these patients. All photographs were taken during breath hold after deep inspiration, with the head of the patients fixed by ear rods. All photographs were digital images, and we used the attached analysis tools on a QXGA 2048 × 1536 dot display (Windows, release 2.8.27NT2; CIS-Image/Viewer IBM Japan Co., Tokyo, Japan) for cephalometric measurements such as direct distance, angle, and area. The direct author who was trained in the measurement methods analyzed all data. The measured sites were as follows: facial axis (FX) and jaw area as skeletal morphology; soft palate area and tongue area as soft tissue morphology; MP-H as mixed morphology (Fig. 1). Tongue area was cross-sectional area outlined by the spina mentalis. JS ratio was obtained from the following formula: JS ratio = (soft palate area + tongue area)/jaw area. We traced the soft tissue outlines, which correspond to the pharyngeal wall, soft palate, and the base of tongue–vallecula. Skeletal discrepancies were evaluated with reference to the cranial base. This is a plane drawn from the anterior nasal spine to the PNS of patient in a natural head position.

### Statistical analysis

All data are presented as mean ± SD. Statistical differences among the groups were analyzed using the Kruskal–Wallis test and the Mann–Whitney test with Bonferroni correction was used for post-hoc test. We used Spearman's rank correlation analysis to estimate the univariate relationship between two variables. To assess the independent association between severe OSAHS (AHI ≥ 30) and cephalometric variables, a logistic regression analysis was used. A forward selection method using likelihood ratio test was selected. Age and BMI were categorized ≥50, ≥25 respectively, and JS ratio was categorized ≥78%. The Hosmer–Lemeshow test was not significant in validating the model. All statistical analyses were performed on a personal computer with the statistical software package SPSS (Windows, release 11.0; SPSS Japan Inc., Tokyo, Japan). A *P*-value of <0.05 was considered to indicate statistical significance.

## RESULTS

### Subject characteristics

The patients were divided into two groups: 107 subjects with mild to moderate OSAHS (5 < AHI < 30) and 95 subjects with severe OSAHS (AHI ≥ 30). Table 1 shows the demographic, polysomonographic and cephalometric characteristics of the patients. The mean age and BMI were significantly greater, and associated with apnea severity. In skeletal morphology, jaw area showed no significant difference among these groups. In soft tissue morphology, soft palate area became greater with severity of OSAHS, and tongue area was greater in severe OSAHS than control. In mixed morphology, MP-H and JS ratio were greater in severe OSAHS than control and 5 < AHI < 30 group (*P* < 0.01).

**Table 1:** Comparison of demographic, polysomnographic and cephalometric variables in patients with OSAHS and control

	Control (*n* = 30)	5 < AHI < 30 (*n* = 107)	AHI ≥ 30 (*n* = 95)	*P*-value
Age (years)	38.3 ± 14.1	46.2 ± 14.6^a^	51.8 ± 12.1^a^	<0.001
BMI (kg/m^2^)	23.3 ± 3.0	26.1 ± 4.5^a^	26.1 ± 4.3^a^	0.003
Neck (cm)	37.6 ± 2.6	38.9 ± 3.2	39.2 ± 2.9^a^	0.098
AHI (events/h)	2.6 ± 1.7	18.0 ± 7.8^a^	61.1 ± 23.8^a^,^b^	<0.001
Lowest oxygen saturation (%)	90.3 ± 2.8	85.1 ± 7.3^a^	79.5 ± 10.7^a^,^b^	<0.001
Skeletal morphology				
FX (°)	84.7 ± 4.1	84.2 ± 4.1	82.7 ± 5.1	0.081
Jaw area (cm^2^)	52.5 ± 5.7	55.3 ± 5.7	53.8 ± 6.6	0.032
Soft tissue morphology				
Soft palate area (cm^2^)	3.6 ± 0.9	4.0 ± 0.8^a^	4.6 ± 1.1^a^,^b^	<0.001
Tongue area (cm^2^)	35.2 ± 4.3	37.2 ± 4.1	38.2 ± 4.6^a^	0.007
Mixed morphology				
MP-H (mm)	18.4 ± 7.1	19.5 ± 5.7	23.7 ± 6.5^a^,^b^	<0.001
JS ratio (%)	73.9 ± 6.5	74.8 ± 7.2	80.0 ± 8.4^a^,^b^	<0.001

Data are mean ± SD.

a *P* < 0.05 *versus* control.

b *P* < 0.05 *versus* mild-moderate OSAHS (5 < AHI < 30). AHI, apnea–hypopnea index; BMI, body mass index; FX, facial axis; JS ratio, jaw soft tissue ratio; MP-H, mandibular plane – hyoid bone; OSAHS, obstructive sleep apnea–hypopnea syndrome.

### AHI and cephalometric variables

Table 2 shows the correlations between AHI and demographic or cephalometric variables in OSAHS patients. Age and BMI were significantly correlated with AHI (*P* < 0.001). In skeletal morphology, FX and jaw area showed no significant correlation with AHI. In soft tissue morphology, soft palate area was positively correlated with AHI. In mixed morphology, MP-H and the JS ratio were positively correlated with AHI (*P* < 0.001).

**Table 2:** The relationship between AHI and demographic or cephalometric variables in patients with OSAHS

	*r*	*P*-value
Age	0.292	<0.001
BMI	0.260	<0.001
Skeletal morphology		
FX	−0.141	0.037
Jaw area	−0.161	0.022
Soft tissue morphology		
Soft palate area	0.326	<0.001
Tongue area	0.105	0.138
Mixed morphology		
MP-H	0.292	<0.001
JS ratio	0.332	<0.001

### BMI and cephalometric variables

Table 3 shows the correlations between BMI and cephalometric variables in OSAHS patients. FX, jaw area, and tongue area were positively correlated with BMI.

**Table 3:** The relationship between BMI and cephalometric variables in patients with OSAHS

	*r*	*P*-value
Skeletal morphology		
FX	0.293	<0.001
Jaw area	0.310	<0.001
Soft tissue morphology		
Soft palate area	0.197	0.005
Tongue area	0.490	<0.001
Mixed morphology		
MP-H	0.060	0.393
JS ratio	0.150	0.034

### Variables for explaining severe OSAHS

Table 4 shows the logistic regression analysis revealed that BMI, age, FX, soft palate area, tongue area, MP-H, and JS ratio are significantly associated with an increased risk for severe OSAHS. After adjusting for BMI and age, these cephalometric variables were significant (Table 4). Jaw area became significant after adjustment. The odds ratio for JS ratio was 2.95 (*P* < 0.001, sensitivity 60.0%, specificity 70.0%), the greatest among these variables.

**Table 4:** Odds ratio of cephalometric variables in patients with severe OSAHS

	OR (95% CI)	Adjust OR (95% CI)
BMI (≥25, kg/m^2^)	2.44 (1.37–4.37)	
Age (≥50, year)	2.03 (1.16–3.57)	
Skeletal morphology		
FX (°)	0.92 (0.86–0.98)	0.88 (0.82–0.95)
Jaw area (cm^2^)	0.96 (0.92–1.01)	0.94 (0.89–0.99)
Soft tissue morphology		
Soft palate area (cm^2^)	2.22 (1.58–3.12)	2.09 (1.45–3.01)
Tongue area (cm^2^)	1.09 (1.01–1.17)	1.05 (0.97–1.14)
Mixed morphology		
MP-H (mm)	1.14 (1.08–1.20)	1.13 (1.07–1.19)
JS ratio (≥78, %)	3.37 (1.89–6.01)	2.95 (1.62–5.37)

Adjusted for age and BMI. CI, confidence interval; OR, odds ratio.

## DISCUSSION

This is the first study showing that JS ratio may be useful for the risk assessment of OSAHS in the Japanese male population based on cephalometric analysis. JS ratio is a newly defined index of mixed morphology presenting a ratio of tongue to soft palate area and jaw area. We found that JS ratio is correlated with AHI, and is a risk factor of OSAHS independent of age and BMI. Both craniofacial and soft tissue morphological anomalies play important roles in the pathogenesis of sleep apnea.

In this study, we found that mixed morphology is intimately related to the severity of OSAHS. Mixed morphology is a combined morphology representing the balance of skeletal morphology such as jaw size, and soft tissue morphology such as tongue or soft palate. The jaw has the role of containing the pharynx, tonsils, fat tissue, and muscles. If the jaw is small, upper airway size becomes narrower despite the same amount of soft tissue.

Previous studies using single morphology have suggested the following parameters as risk indicators of OSAHS: small jaw,^2^,^6^,^9^ long pharynx,^14^ retrognathia,^6^,^11^ and decreased facial axis^25^,^26^ in skeletal morphology; and increased soft palate and tongue area^10^,^11^,^27^ in soft tissue morphology. Another study showed that tongue volume is correlated with BMI,^19^ which can identify the risk factor of OSAHS with obesity. Our results are consistent with these studies, except for tongue area. In our study, correlation was found with BMI.

In the Japanese population, 29% of non-obese patients (BMI < 25) and 45% of mildly obese patients (25 ≤ BMI < 30) have an AHI greater than 20.^28^ Our study included 44.6% non-obese patients. Most of the patients were thus considered to have a high risk of OSAHS associated with their craniofacial morphology, independent of obesity. In contrast to Caucasians, whose craniofacial structures are developed horizontally, craniofacial structures of Asians are developed vertically. This characteristic of Asians is called “long face” and is causally related to increased total and upper facial heights compared with those of Caucasians.^10^ The upper airways of Asians tend to collapse more easily than those of Caucasians with the same body size and fat distribution.^29^ This may be a major reason for having more severe sleep apnea in Asians compared with Caucasians with the same BMI. Asians have combined risks of craniofacial structure and obesity,^9^,^10^ therefore mixed morphology measurement may be useful for OSAHS diagnosis in Asian population.

Several studies concerning mixed morphology have been conducted. Tuiki *et al*.^20^ matched the craniofacial dimension and BMI in OSA and non-OSA subjects and showed that OSA patients had larger tongue size and longer MP-H distance. Watanabe *et al*.^11^ evaluated the influence of BMI and craniofacial anomalies, and reported that sleep disorder breathing patients with positive closing pressures at both the velopharynx and oropharynx had longer MP-H distance compared to a non-OSAHS group.

A significant value of JS ratio also may be an important indicator because the ratio represents the balance between the jaw area as a container and the soft palate and tongue area as its content. All these parameters showed a significant but weak correlation with AHI. In the studies examining the ratio of the oral cavity capacity and the tongue area or soft palate area, Tangugsorn *et al*.^18^ evaluated the tongue area and soft palate area relative to the oral and pharyngeal area using cephalometry. The ratio was significantly higher in both non-obese and obese OSAHS patients compared to control subjects. Lowe *et al*.^17^ evaluated the tongue volume relative to the oral cavity volume using three-dimensional CT, and showed the correlation with AHI. Kondo *et al*.^19^ performed a similar evaluation using MRI. These indices can be measured precisely by CT or MRI analysis; however, cephalometry is a cost effective method using a single X-ray photograph.

In our study, we observed a significant but weak correlation with AHI in all mixed morphologies including JS ratio and MP-H. An elongation of MP-H however does not always indicate upper airway size. Increase of MP-H is attributed to narrowing of the mandible or increase of the parapharyngeal adipose tissue,^30^,^31^ and may result in shifting the hyoid bone caudally. A downward shift of the hyoid bone can happen to individuals who have small jaw area with normal size of tongue and upper airway. In addition, both indices are significant explanatory variables explaining AHI ≥ 30 by logistic regression analysis even after adjustment for age and BMI, with the odds ratio of JS ratio greater than MP-H. We thus conclude that JS ratio may be an appropriate variable to evaluate the risk of OSAHS representing balance between skeletal morphology and soft tissue morphology whereas MP-H may be more affected by skeletal morphology.

### Limitations

This study has some limitations. First, only Japanese patients were included. Although there are differences in frequency of OSAHS between races, craniofacial morphologic risk is a common problem in all races. If the influences of age or BMI are excluded, similar results may be obtained for other races. Second, only male patients are included in this study. Gender difference in OSAHS^5^,^6^ is well known and different results may be obtained with the inclusion of female patients. Third, as cephalometry was performed in a sitting position while awake, the results of this study may not represent the upper airway in a supine position during sleep. However, previous reports using cephalometry have provided a significant association between AHI and measurement variables.^11^,^20^ Fourth, 30 subjects whose AHI < 5 were selected as control; however, these subjects may not be healthy volunteers since they were symptomatic. This selection bias could affect the results of this study. Fifth, we excluded tonsillar hypertrophy from patient selection, as large tonsillar hypertrophy is known as a risk for OSAHS even in lean patients with normal airways. Since the degree of hypertrophy is different among individual patients, we thought if patients with tonsillar hypertrophy were included in this study, the results would be confused and become unclear. The authors aimed to find the role of the soft palate and tongue as soft tissue in this study, and thus excluded tonsillar hypertrophy.

## CONCLUSIONS

The results of this study showed that mixed craniofacial skeletal and soft tissue morphology are significantly correlated with AHI. JS ratio was one of the variables significantly associated with an increased risk for severe OSAHS by logistic regression analysis, remaining significant even after adjusting for age and BMI. Its odds ratio was greatest among these variables. The results suggest that craniofacial morphology evaluation is a clinically valid method for explaining the characteristics of severe OSAHS in male patients, and JS ratio is a useful parameters to identify risk factors of OSAHS.
